# Equipping the next generation: addressing the unmet need for artificial intelligence education at research performing organizations

**DOI:** 10.1186/s41073-026-00250-x

**Published:** 2026-10-16

**Authors:** Jeremy Y. Ng, Wid Al-Zahraa Al-Khafaji, David Moher

**Affiliations:** 1 Institute of General Practice and Interprofessional Care, University Hospital Tübingen, Osianderstr. 5, Tübingen, 72076 Germany; 2 Robert Bosch Center for Integrative Medicine and Health, Bosch Health Campus, Stuttgart, Germany; 3 Department of Health Research Methods, Evidence, and Impact, Faculty of Health Sciences, McMaster University, Hamilton, Canada; 4 School of Public Health, Faculty of Health, University of Technology Sydney, Sydney, Australia; 5 Centre for Journalology, Ottawa Methods Centre, Ottawa Hospital Research Institute, Ottawa, Canada; 6 School of Epidemiology and Public Health, University of Ottawa, Ottawa, Canada; 7 Institute of Health Policy, Management & Evaluation, Dalla Lana School of Public Health, University of Toronto, Toronto, Canada

**Keywords:** Artificial intelligence chatbots, Research integrity, Higher education, AI literacy, Medical and life sciences education, Students, Research performing organizations

## Abstract

**Background:**

The integration of generative artificial intelligence (GenAI) tools into higher education is rapidly accelerating, yet the educational ecosystem appears largely unprepared for the scale and speed of adoption. Our recent international survey of over 1,200 university students and postdoctoral fellows in the medical and life sciences found that while more than 80% had used GenAI tools, the vast majority had received no formal training, and many were unaware of any institutional policies governing their use. This gap between adoption and institutional preparedness may pose risks to research integrity if left unaddressed.

**Main body:**

Existing research supports a coordinated, evidence-informed approach to AI education in research performing organizations (RPOs), grounded in principles of research integrity, equity, and critical engagement with technology. First, GenAI literacy must be embedded as a core competency in scientific and clinical education, equipping students to critically evaluate tool outputs, recognize hallucination and bias, and understand the distinction between AI-generated inaccuracy and deliberate research misconduct. Second, traditional frameworks for academic integrity would benefit from re-examination. Today, GenAI tools have fundamentally disrupted established notions of authorship, originality, and plagiarism, and the absence of coherent institutional guidance leaves students and educators navigating these questions without support. Third, RPOs must develop clear, transparent, and context-sensitive policies on GenAI use by embedding responsible AI education into curricula, equipping learners not only to use these tools effectively, but to question, verify, and critique their outputs in ways that uphold scientific standards.

**Conclusion:**

RPOs have an urgent responsibility to move beyond reactive responses and toward proactive strategies that uphold scientific standards, promote equity of access, and prepare the next generation of researchers and clinicians to engage with GenAI responsibly and ethically.

## Introduction

Artificial intelligence (AI), particularly in the form of generative artificial intelligence (GenAI) tools such as OpenAI’s ChatGPT, Google Gemini (formerly Google Bard), Perplexity, Meta AI, Claude AI, and Microsoft Copilot (formerly Bing Chat), is transforming how knowledge is accessed, synthesized, and communicated across academic settings. These tools are no longer confined to computer science or engineering departments; rather, they have rapidly permeated disciplines as diverse as medicine, life sciences, education, law, and the humanities. In higher education, GenAI tools offer both undergraduate and graduate students and postdoctoral fellows the ability to quickly summarize complex topics, generate text-based outputs, translate content, troubleshoot technical problems, or ideate in real time, tasks that traditionally required extensive human input, time, and expertise. The allure of speed, convenience, and round-the-clock availability has made free-to-use GenAI tools appealing to university students and postdoctoral fellows,navigating the pressures of increasingly demanding academic environments [1, 2].

However, this technological evolution presents both immense opportunities and sobering challenges. On the one hand, GenAI tools have the potential to enhance productivity, personalize learning, and expand access to academic support, particularly for university students and postdoctoral fellows who may face structural barriers such as limited instructor availability, language difficulties, or lack of access to tutoring services [3–5]. On the other hand, their widespread and unregulated use raises serious concerns related to misinformation, overreliance, research integrity, and unequal access [6–8]. Yet despite the benefits of GenAI, access is not universal: while basic-tier GenAI tools are freely available at no cost, meaningful access requires reliable internet connectivity; a resource that remains unavailable or unaffordable for a significant proportion of students in low- and middle-income countries (LMICs) [7, 9]. Today, only about 29%−60% of people living in LMICs have access to the internet, that is an estimate of 2.2 billion people that remain offline and without access to GenAI tools [9, 10]. Furthermore, the most capable versions of these tools increasingly require paid subscriptions, raising concerns about a new form of digital inequity in which students and institutions in under-resourced settings, particularly in the Global South, may be systematically excluded from the full benefits of these technologies [4, 7, 11, 12]. Institutions should pursue resource-sensitive implementation strategies, such as open educational resources, training modules, and shared training consortia, to avoid GenAI literacy mandates that themselves widen digital inequity [13, 14]. As GenAI becomes more integrated into the academic workflow across all stages of learning and research, including literature searching and hypothesis generation to writing, peer review, and knowledge dissemination questions about authorship, originality, and the very nature of learning are being fundamentally redefined [15–17].

Despite the growing reliance on these tools, the educational ecosystem appears largely unprepared for the scale and speed at which GenAI tools are being adopted by students. In our recent large-scale, international survey of 1,209 university students and postdoctoral fellows in the medical and life sciences, we found that while more than 80% of respondents had used GenAI tools, the vast majority had received no formal training [18]. This cross-sectional survey recruited participants internationally through two channels, including corresponding authors of recent MEDLINE-indexed publications, and program administrators at globally ranked, English-language universities; therefore reflecting a self-selected, English-proficient, research-active sample that may not generalize to all university students and postdoctoral fellows, disciplines, or world regions [18]. Furthermore, many were unaware of any institutional policies regulating such use, and where policies did exist, they were often unclear, inconsistently applied, or poorly communicated [18]. This gap between technological adoption and institutional preparedness signals an urgent need for research performing organizations (RPOs) to catch up, not only by offering technical guidance, but by fostering critical literacy, ethical reasoning, and evidence-informed practice in the use of GenAI.

The issue is not simply that students and postdoctoral fellows are using GenAI tools, but that they are doing so in the absence of structured support at the institutional level [18, 19]. As a result, students are often left to navigate a complex landscape of emerging technologies without clear direction, increasing the risk of misuse, inequity, and erosion of academic standards [6, 20].

This commentary draws on the findings of our survey to explore the implications of unstructured GenAI tool use in higher education. We argue that RPOs such as universities, academic medical centres, and research institutes, must move beyond reactive, punitive responses and toward proactive strategies that equip university students and postdoctoral fellows with the tools to engage responsibly with GenAI technologies [20]. In doing so, we offer a roadmap for how RPOs can respond thoughtfully and equitably to this inflection point in educational transformation. While RPOs encompass a broad range of institutions, including universities, academic medical centres, and research institutes, the evidence and recommendations offered in this commentary are grounded primarily in university-based biomedical and life sciences environments; RPOs with different goals, learner populations, or resource levels may need to adapt these recommendations to their own context.

Although interconnected, GenAI literacy, academic integrity, and responsible conduct of research (RCR) represent related but distinct educational needs. GenAI literacy concerns the skills required to understand, critically evaluate, and appropriately use GenAI systems, including recognizing their capabilities, limitations, and biases [21]. Academic integrity concerns the norms of authorship, attribution, originality, and assessment that govern individual coursework and scholarly outputs [22]. RCR, by contrast, encompasses the broader ethical norms governing the research enterprise as a whole, including data integrity, reproducibility, conflicts of interest, and the definition and prevention of research misconduct [23, 24]. RPOs should ensure GenAI education addresses all three domains explicitly, rather than treating GenAI literacy as a subset of academic integrity training alone.

## The normalization of GenAI tools in research performing organizations: how use outpaces policy

Our survey found that 63% of respondents were already familiar with GenAI tools, and nearly 76% were either likely or very likely to use them in the future [18]. ChatGPT, by far the most popular tool reported in the aforementioned survey, was routinely used for learning new topics, simplifying complex concepts, and supporting administrative tasks like email drafting and scheduling. Yet this widespread adoption, in our view, is happening in a regulatory and pedagogical vacuum. Over 56% of respondents stated that their institutions had not formally integrated GenAI tools into educational practices, and 59% reported that no training had been offered. Nearly half were unsure if any policies even existed [18]. This mismatch between practice and policy signals a critical oversight.

## AI literacy as a core competency in clinical and scientific education

Having shown that GenAI adoption is outpacing institutional policy, we turn first to the need to embed GenAI literacy as a core competency in scientific and clinical education. For university students and postdoctoral fellows in the life and medical sciences, fields in which precision, accountability, and evidence-based reasoning are not only valued but essential, the stakes surrounding the use of GenAI tools are particularly high [25, 26]. These learners are preparing for careers where errors can result in harm to patients, misallocation of resources, or erosion of public trust in science. As such, their use of GenAI tools to assist with research writing, interpret clinical or laboratory data, generate hypotheses, or summarize published evidence must be grounded in a deep understanding of how these tools function, where they fail, and how to use them responsibly.

Using GenAI tools effectively in academic and clinical contexts requires far more than technical familiarity, it demands critical insight and discernment: the capacity to evaluate the truth-value, reliability, and origin of information. Without formal instruction in GenAI literacy, university students and postdoctoral fellows may lack the tools to assess the quality of chatbot outputs, distinguish between credible and spurious information, or recognize when they are unknowingly misapplying technology to inappropriate or high-stakes contexts [27]. Without the training to verify sources and critically appraise content, RPOs risk incorporating factually inaccurate or fabricated information into their academic work. This risk is conceptually distinct from traditional research misconduct, and is defined under established frameworks as fabrication, falsification, and plagiarism (FFP), as AI-generated inaccuracies typically arise not from deliberate intent to deceive, but from the probabilistic nature of large language model (LLM) outputs: a phenomenon known as hallucination, in which generated text appears coherent and plausible but is factually incorrect or unsupported by evidence [23, 28–30]. For example, a student relying on ChatGPT to generate a systematic review may receive synthesized content that omits key evidence, misrepresents study findings, or fabricates citations [31]. Bibliographic hallucinations, in which LLMs produce fabricated or partially incorrect citations, are of particular concern in the medical and life sciences, where the verifiability of cited evidence is fundamental to scientific integrity [32]. The problem is compounded by a systemic gap in research training: while funders such as the National Institutes of Health (NIH) mandate RCR instruction for NIH-supported trainees, this requirement applies only to those receiving NIH funding and does not extend universally to all students and staff at RPOs [24]. As a result, a significant proportion of students using GenAI tools may never have received formal instruction in research integrity, and are therefore poorly equipped to recognize where AI-generated content crosses into the territory of misinformation, research misconduct, or unintentional fabrication.

The consequences of such missteps are not trivial. In medicine and life sciences, flawed data interpretation or misleading summaries can influence clinical decisions, public health recommendations, or future research priorities [33]. These fields require a higher standard of verification, reproducibility, and traceability than many GenAI tools are currently capable of providing [34]. Thus, a laissez-faire approach to GenAI use in scientific training may be inadequate, and in high-stakes contexts, potentially unsafe [35].

To address these challenges, GenAI literacy must be integrated as a core competency within modern scientific and clinical education [36]. This goes beyond simply knowing how to use GenAI tools, it includes fostering critical awareness, reflective judgment, and ethical responsibility. Essential elements of a GenAI literacy curriculum should include:

- Understanding how GenAI models are developed, trained, and optimized, including basic knowledge of natural language processing, large language models, and dataset limitations.
- Recognizing the presence of bias, uncertainty, and hallucination in GenAI-generated outputs, especially in relation to underrepresented populations, controversial topics, or specialized scientific domains [26].
- Maintaining academic integrity in the use of GenAI tools, including understanding what constitutes appropriate versus inappropriate use in different educational contexts (e.g., brainstorming vs. ghostwriting).
- Learning when and how to cite GenAI-generated content, where permitted by institutional policies, and understanding that attribution does not absolve critical responsibility.
- Developing the habit of verifying GenAI-generated claims against primary literature, structured databases, or domain experts, especially when using GenAI tools to support decision-making or scholarship [31].
- Using GenAI tools to augment human thinking, for example, to support ideation, feedback, or knowledge retrieval, rather than as replacements for original analysis, synthesis, or argumentation.

Equally important is the recognition that GenAI tools are not neutral technologies. They are shaped by the data on which they are trained, the goals they are optimized to achieve, and the institutions and companies that build them [37]. University students and postdoctoral fellows must be taught to interrogate, rather than blindly trust, GenAI-generated outputs [38]. This includes asking whose voices are amplified or erased by these systems, what assumptions are embedded in their design, and what harms may arise from their use.

Embedding GenAI literacy into the curriculum also creates opportunities for interdisciplinary dialogue. Courses that address the intersections of machine learning, ethics, information science, and subject-specific knowledge can better prepare university students and postdoctoral fellows to work in complex environments where data, technology, and human judgment co-exist [36, 39]. Just as statistical literacy and research ethics have become standard components of scientific training, so too must GenAI literacy evolve into a foundational skill for all future scientists and clinicians [40].

## Reimagining academic integrity in the age of AI

Beyond literacy, RPOs face a second priority: reconsidering how conventional academic-integrity frameworks apply in an environment shaped by GenAI. Academic integrity has long been a cornerstone of higher education, reflecting the values of honesty, responsibility, and respect for intellectual labour. It underpins trust between students and instructors, among peers, and in the academic system as a whole. Yet the emergence of GenAI tools has disrupted traditional notions of authorship, originality, and plagiarism, prompting a necessary re-examination of what academic integrity means in the age of generative AI [17, 41, 42].

GenAI tools like ChatGPT can now generate coherent, persuasive, and grammatically correct content in seconds. They can summarize academic texts, propose thesis statements, rewrite passages in different tones, and even generate complete essays with relatively minimal input. This raises complex ethical questions: Is it unethical for a student to use ChatGPT to generate a first draft of an essay if they subsequently revise and substantively engage with it? Should GenAI-generated summaries be cited, and if so, how? What constitutes "original" work if the content is produced by a machine trained on vast swaths of publicly available text? Can instructors ethically mandate or prohibit the use of GenAI tools without providing adequate alternatives or training?

Our survey revealed that university students and postdoctoral fellows experience a wide range of responses to these questions depending on the instructor, course, or institution [18]. Some respondents reported that their instructors explicitly encouraged GenAI tool use, with the condition that any use be disclosed and used only for early-stage ideation or writing support. Others encountered blanket prohibitions on GenAI use, including in contexts where it may have been appropriate or beneficial. Many university students and postdoctoral fellows indicated they were unsure of the rules altogether, with no consistent messaging from their institutions about what was permissible [18]. This lack of clarity understandably is creating a confusing learning environment for both students and instructors alike.

This ambiguity is not merely a procedural issue, it carries serious pedagogical and ethical implications. When expectations around GenAI use are unclear or inconsistently applied, students are left to make their own interpretations, often in high-stakes settings such as exams, grant proposals, or thesis submissions [41]. Some may overuse GenAI tools out of ignorance, others may underuse them or resist learning about them out of fear, and still others may intentionally exploit the lack of oversight [42]. In all cases, the absence of coherent institutional guidance threatens to erode trust in the educational process and widen existing disparities between university students and postdoctoral fellows with different levels of digital literacy, institutional support, or access to GenAI tools [39].

## Institutional responsibilities and the way forward

Translating these literacy and integrity considerations into practice requires the third and final priority: institutional action. RPOs cannot treat AI as a passing trend or peripheral skill. The findings from our survey make it clear: university students and postdoctoral fellows are already using these tools, and they want guidance. Institutions must meet this demand with structured, evidence-based GenAI education embedded across disciplines and programs.

To address this, RPOs must urgently develop and implement clear, transparent, and context-sensitive guidelines on GenAI tool use [19, 36, 43]. These guidelines should:

- Define acceptable and unacceptable use of GenAI tools in a range of academic contexts (e.g., coursework, exams, research, thesis writing), while recognizing disciplinary differences and levels of academic maturity [19].
- Include university students and postdoctoral fellows as active partners in the policy-making process to ensure that rules are informed by the real-world challenges and practices of those most affected [44].
- Explicitly distinguish between GenAI use that supports learning (e.g., using GenAI tools to brainstorm, summarize feedback, or translate content) and GenAI use that undermines academic integrity (e.g., submitting GenAI-generated work without meaningful engagement or attribution) [36, 43].
- Promote transparency and disclosure as standard practice, encouraging university students and postdoctoral fellows to declare when and how GenAI tools were used, in the same way that reference managers or grammar tools are disclosed when required [39].

Students and postdoctoral fellows cannot be expected to use GenAI responsibly if their supervisors and instructors lack guidance, RPO-level GenAI education should extend to faculty and research mentors, not only learners [45]. Table 1 outlines how RPOs might sequence this work, from immediate interim guidance through to longer-term governance.

**Table 1:** A Roadmap for RPO Implementation of GenAI Education and Governance

Timeframe	Priority action	Lead responsible actor(s)	Illustrative evaluation indicator
Immediate (0–6 months)	Issue interim guidance on permissible GenAI use in coursework, assessments, and research writing, including disclosure expectations	Research integrity office; teaching and learning centre	Proportion of students/staff aware of institutional GenAI policy
Medium-term (6–18 months)	Embed GenAI literacy modules into research methods, RCR, and clinical/scientific curricula; launch faculty development programming	Curriculum committees; programme directors; faculty developers	Course completion rates; pre/post GenAI literacy assessment scores
Long-term (18 + months)	Establish institution-wide GenAI governance, including equity-sensitive access provisions and periodic policy review	Senior leadership; IT services; student/postdoc representatives	Annual audit of GenAI access, training uptake, and reported integrity concerns

Timeframes are illustrative and intended as a starting point; institutions should adapt them to their own governance processes and readiness

Equally important is the need to shift from a punitive to an educational mindset. Rather than defaulting to disciplinary action when students misuse GenAI tools, institutions should treat these incidents as opportunities for learning. Many university students and postdoctoral fellows are still discovering the capabilities and limitations of GenAI tools and may not yet understand the ethical boundaries of their use. Educators should create space for reflection, discussion, and guidance, integrating conversations about AI ethics and digital responsibility into the curriculum itself.

Institutions must also rethink traditional assessment strategies, which are increasingly vulnerable to GenAI-driven short-cuts. Assignments that can be easily completed by GenAI tools, such as generic essays, take-home quizzes, or unmonitored reflections, no longer reliably measure student understanding. Instead, educators might adopt:

- Oral assessments or viva-style defenses where university students and postdoctoral fellows must explain their reasoning and process [46].
- In-class writing exercises, which provide controlled environments for authentic demonstration of knowledge [47].
- Iterative assignments that require submission of drafts, peer feedback, and reflective commentary on the development process [48].
- Portfolio-based assessments, in which university students and postdoctoral fellows curate evidence of learning over time, supported by documented process work and personal reflection [49].

By aligning assessments with process-oriented, analytical, and higher-order thinking skills, educators can reduce opportunities for unethical GenAI use while simultaneously deepening learning.

## Limitations and future directions

This commentary’s limitations include that it is not an original empirical study but a perspective grounded substantially in a single survey of medical and life sciences university students and postdoctoral fellows; as noted above, the self-selected and English-proficient nature of that sample means findings may not generalize to other disciplines, career stages, or world regions [18]. As this is a commentary rather than a systematic or scoping review, the supporting literature was selected to illustrate key arguments rather than to comprehensively map the field. Future research should examine GenAI literacy, policy, and educational needs across broader and more diverse RPO populations, including non-English-speaking institutions, non-biomedical disciplines, and RPOs in LMICs, and should evaluate the effectiveness of specific GenAI education interventions using pre/post or longitudinal designs.

## Conclusion

Reimagining academic integrity in the age of GenAI requires a more nuanced and forward-thinking approach, one that acknowledges the transformative potential of these tools while safeguarding the values of transparency, rigour, and fairness that are foundational to research integrity. As GenAI becomes increasingly embedded in both academic and professional settings, preparing university students and postdoctoral fellows to engage with it ethically and skillfully is not just a pedagogical responsibility; it is an obligation to the integrity of science itself. RPOs are presently faced with a critical imperative: to empower the next generation of researchers and clinicians with the tools, language, and ethical grounding needed to navigate GenAI's promises and perils. GenAI tools will not, and should not, replace educators or researchers. But institutions that fail to engage seriously with these tools risk producing graduates who are neither prepared nor accountable in their use. As GenAI tools, risks, and capabilities are evolving rapidly, RPO policies and curricula should require periodic review rather than one-time implementation. By treating GenAI education as a foundational element of research training, RPOs can ensure that university students and postdoctoral fellows are not only proficient users of GenAI, but also critical thinkers, ethical scholars, and future-ready professionals capable of upholding the standards that science demands.

## Data Availability

No datasets were generated or analysed during the current study.

## Abbreviations

AI: Artificial intelligence
FFP: Fabrication, falsification, and plagiarism
GenAI: Generative artificial intelligence
LLM: Large language model
LMICs: Low- and middle-income countries
RCR: Responsible conduct of research
RPO: Research performing organization
